# Associations Between Eye-Movement Patterns, Pupil Dynamics, and the Interpretation of a Single Mixed-Dentition Panoramic Radiograph Among Dental Students: An Exploratory Eye-Tracking Study

**DOI:** 10.3390/vision10010013

**Published:** 2026-02-14

**Authors:** Satoshi Tanaka, Hiroyuki Karibe, Yuichi Kato, Ayuko Okamoto, Tsuneo Sekimoto

**Affiliations:** 1 Department of Pediatric Dentistry, School of Life Dentistry at Tokyo, The Nippon Dental University, Tokyo 102-8159, Japan; h-karibe@tky.ndu.ac.jp (H.K.); ykato@tky.ndu.ac.jp (Y.K.); okamotoa@tky.ndu.ac.jp (A.O.); 2 The Nippon Dental University, Tokyo 102-8159, Japan

**Keywords:** eye-tracking, pupillometry, panoramic radiography, mixed dentition, visual search, fixation, saccades, cognitive load, dental students, radiographic interpretation

## Abstract

Eye tracking can provide quantitative indices of visual exploration and cognitive processing during radiographic image interpretation. This study examined eye-movement patterns and pupil dynamics and their associations with task performance while fifth-year dental students interpreted a single mixed-dentition panoramic radiograph under free-viewing conditions. Task performance was defined as the number of correctly identified pre-specified items (three radiographic findings plus two interpretive items: dental age estimation and the presence/absence of congenital anomalies). Eye-movement patterns were classified into four groups: clockwise (R, 29.6%), counterclockwise (L, 44.4%), saccadic (S, 16.7%), and concentrated (C, 9.3%). Clockwise scan paths were associated with higher task scores and more globally distributed fixations than other patterns (*p* < 0.001). Linear mixed-effects modeling suggested that task scores increased up to 120 s of viewing time, whereas longer viewing times were not associated with further improvements. Furthermore, ordinal logistic regression analysis revealed that higher task scores were significantly associated with a smaller mean pupil area across the entire viewing time, combined with a larger pupil area specifically during fixations, suggesting more selective allocation of cognitive resources. These findings indicate associations between global scan structure, time allocation, pupil dynamics, and task performance in this single-image setting. Generalization to overall diagnostic competence or other radiographs requires replication using multiple panoramic images and a broader range of verified findings.

## 1. Introduction

Research on visual processing has rapidly expanded across multiple domains, ranging from computer vision and deep learning to improving diagnostic imaging and surgical precision [1], using virtual and augmented reality in dental education [2], integrating vision and language [3], and investigating eye-tracking and attentional mechanisms [4].

In particular, eye-tracking studies have provided valuable insights into human cognition and information processing. In cognitive psychology, eye tracking has been used to examine how individuals perceive and process information, including the analysis of face-viewing patterns [5], the relationship between cognitive load and attention [6], learning processes such as the improvement of dyslexia through visual attention training [7], and associations between dyslexia and impaired visual attention [8].

In the dental field, we previously applied eye tracking to investigate how undergraduate dental students process panoramic radiographs (PANs) of mixed dentition [9]. We demonstrated that eye movement patterns can be classified into three categories associated with task performance. High-scoring students exhibited significantly larger pupil diameters, suggesting greater engagement in the tasks. However, pupil dilation is also known to reflect increased cognitive load [10]. Castner et al. [11] reported that dental students and radiology experts differ in their cognitive load during PAN interpretation, with experts showing smaller pupil diameters and thus, lower cognitive demands. In our earlier study, the observed pupil dilation among students with higher task scores might have been influenced by the experimental design, which imposed a strict 1 min viewing time limit. This time constraint might have placed the undergraduate students under an unusually high cognitive load, potentially altering their pupillary responses.

Previous research by Gnanasekaran et al. allowed undergraduate dental students to freely observe PANs of mixed dentition without time restrictions and reported their average viewing times [12]. However, their work did not address how pupil dynamics or the duration of viewing time influenced task performance. To date, no study has examined the relationship between eye-movement patterns, pupil responses, and diagnostic outcomes during free-viewing of mixed-dentition PAN interpretation.

This study aimed to examine whether visual perceptual patterns remained associated with task performance when time constraints were removed. Dental students were allowed to observe a single mixed-dentition PAN for as long as they wished, and the interpretation ended at their own discretion.

We hypothesized that students with higher task scores would demonstrate (1) a clockwise eye-movement pattern, (2) longer viewing time up to an optimal range, and (3) pupil-response characteristics consistent with more efficient allocation of cognitive resources.

Accordingly, this study aimed to characterize eye-movement patterns and pupil dynamics during the interpretation of a single mixed-dentition PAN and to explore their associations with task performance. These findings may inform future hypothesis-driven studies and the development of educational approaches; however, the present study is designed as an exploratory single-image task and does not aim to establish generalizable training efficacy.

## 2. Materials and Methods

### 2.1. Participants

Fifty-four fifth-year dental students (41 male and 13 female) from the Nippon Dental University School of Life Dentistry at Niigata participated in this study. The purpose and procedures of the study were explained in advance, and written informed consent was obtained from all participants. The study protocol was approved by the Ethics Committee of Nippon Dental University School of Life Dentistry at Niigata (approval no. ECNG-H-106). All participants had normal or corrected-to-normal vision (≥0.8, Landolt ring method with contact lenses permitted) and no conditions that could interfere with the experiment. All participants had passed the nationwide Common Achievement Tests (CBT) required for clinical training, indicating that they had completed fundamental dental education, including pediatric dentistry and oral radiology, and had the baseline knowledge necessary to interpret PANs. The students received unified preclinical training in structured radiographic interpretation before participating in this experiment. This included the identification of congenitally missing teeth, recognition of supernumerary teeth in the midline region, evaluation of tooth germ numbers, assessment of Hellman’s dental age based on eruption status, interpretation of the radiographic appearance of space maintainers, and use of the FDI World Dental Federation notation system as the standard for tooth identification. PANs were interpreted according to the conventional viewing sequence based on standard radiographic instruction (upper right → upper left → lower left → lower right). All classes were conducted by the same instructor, ensuring that the participants received equivalent instruction under identical educational conditions. All students had passed the standardized CBT; therefore, we considered that their baseline competency for PAN interpretation had been sufficiently calibrated and did not perform any additional pre-tests. However, implementing a formal calibration test may further enhance methodological rigor in future studies. We estimated the required sample size a priori for a four-group one-way ANOVA (fixed effects; two-sided; α = 0.05; desired power = 0.80). The effect size (Cohen’s f = 0.85) was derived from our pilot data (group means and SDs), using the pooled within-group SD under equal allocation. The analysis indicated that at least five participants were required per group. Calculations were performed with G*Power (version 3.1.9.7; Heinrich-Heine-Universität Düsseldorf, Düsseldorf, Germany).

### 2.2. Test Image

**Figure 1:**
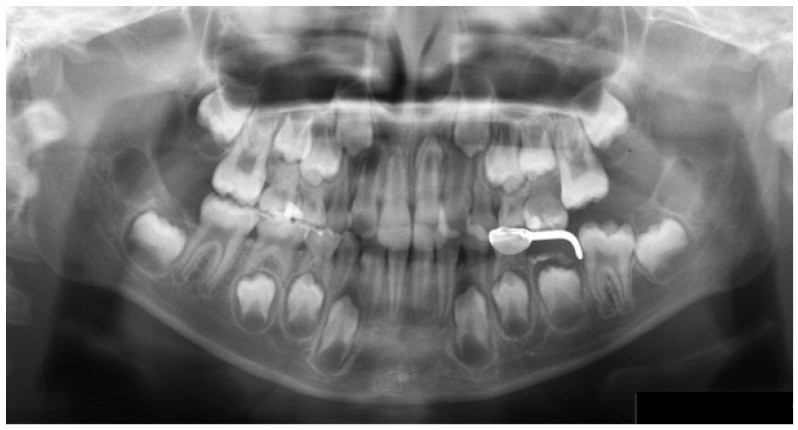
Test image.

A single mixed-dentition PAN (Hellman’s dental age IIIA) was used as the test image (Figure 1). The image contained three predefined radiographic findings used for scoring (an inverted impacted maxillary supernumerary tooth, a space maintainer, and a restoration). In addition, two interpretive items were scored: estimation of dental age (Hellman’s method) and recognition of congenital anomalies as present/absent (absent in this radiograph). These five pre-specified items constituted the task score and were selected to ensure ground-truth verifiability and scoring reliability within a single-image design. Other potential findings (e.g., suspected caries lesions or third-molar-related findings) were not included in the task score because reliable ground-truth verification from this single radiograph alone could not be ensured and consistent scoring would have required additional clinical data (e.g., clinical records or additional views) or radiographic information to confirm the diagnosis. Each participant was instructed to interpret the image and informed that it would be displayed until they indicated that their interpretation was complete. All participants completed radiology coursework and were undergoing clinical training at the time of the study.

### 2.3. Eye Movement Recording

**Figure 2:**
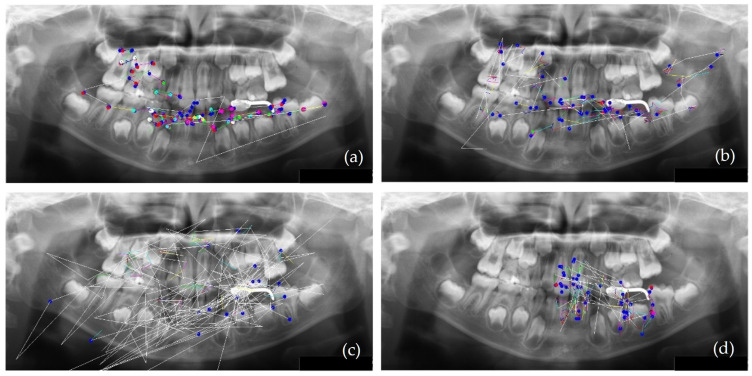
Scan path patterns during PAN interpretation. (**a**) R group (clockwise), (**b**) L group (counterclockwise), (**c**) S group (scattered pattern with sparse fixations and frequent wide-range saccades), and (**d**) C group (clustered fixations around anatomical landmarks with minimal quadrant transitions). Colored dots represent fixation points, and lines show saccades. Fixation colors indicate duration thresholds (blue = 33 ms, red = 66 ms, pink = 100 ms, green = 133 ms, light blue = 166 ms, yellow = 200 ms, white = 233 ms), and line colors indicate saccade velocity (10–70 deg/s in ascending order). Representative scan paths are shown; therefore, these examples do not quantify tooth-by-tooth coverage or AOI-based fixation across all regions.

The PAN was projected onto a screen (106 cm × 141 cm) using a liquid crystal projector (EMP-755, Seiko Epson Corporation, Nagano, Japan). The screen was positioned 200 cm from the participant, subtending a visual angle of approximately ±20° horizontally and ±15° vertically (Figure 2). Eye movements were recorded using a non-contact eye tracker (Free View T.K.K. 2920; Takei Scientific Instruments Co., Ltd., Niigata, Japan). This system tracks the gaze direction by capturing near-infrared reflections from the pupil and corneal Purkinje images, allowing conversion into gaze coordinates. Fixations were defined as gaze points with angular velocity < 5°/s, and saccades as movements ≥ 5°/s. Eye movements were recorded continuously from image onset until participants voluntarily terminated their interpretation.

### 2.4. Assessment of Task Performance

After the eye-tracking session, participants moved to a separate room and freely recorded their interpretations in writing. Five pre-specified items were extracted from the responses: (1) inverted maxillary impacted supernumerary tooth, (2) space maintainer, (3) restoration, (4) dental age (Hellman’s method), and (5) congenital anomalies (present/absent; absent in this radiograph). The number of correctly identified items was converted to a task score. The participants were then categorized into two groups: high-score group (≥3) and low-score group (<3). Task performance was analyzed according to sex, viewing time, and eye-movement patterns. Viewing time was classified into four categories: 0–60 s, 61–120 s, 121–180 s, and ≥181 s.

### 2.5. Eye-Tracking Metrics

Fixation count, total viewing time, mean saccadic velocity, fixation ratio, total fixation duration, and mean fixation duration were calculated. Fixations were defined as gaze maintained for ≥33 ms. With 1818 frames acquired per minute, total viewing time was calculated as the number of valid frames (excluding blink frames) multiplied by 33 ms. Mean saccadic velocity was calculated as total angular displacement divided by total viewing time. The fixation ratio was defined as the number of fixation frames divided by the total number of viewing frames. The total fixation duration was defined as the number of fixation frames multiplied by 33 ms, and the mean fixation duration was defined as the total fixation duration divided by the fixation count.

Blink analysis distinguished between spontaneous blinks (short duration, ≤330 ms, occurring periodically) and Voluntary blinks (longer duration, non-periodic). Rapid serial blinks were defined as clusters of consecutive blinks within a short interval.

Pupil area was analyzed as an index of cognitive load, attention, and mental state. Both the overall pupil (excluding blink frames) and fixation-related pupil areas (excluding saccades, analyzed only during fixations) were calculated. Pupil area was measured in dot units and expressed as π·dot^2^.

### 2.6. Classification of Eye Movement Patterns

Eye movement patterns were classified based on the gaze coordinates (*x*, *y*) sampled every 33 ms. Using three consecutive points, *A* = (*Ax*, *Ay*), *B* = (*Bx*, *By*), and *C* = (*Cx*, *Cy*), the cross-product was calculated as follows:

*S* = (*Bx* − *Ax*)(*Cy* − *Ay*) − (*By* − *Ay*)(*Cx* − *Ax*)

If *S* < 0, the movement is clockwise; if *S* > 0, it is counterclockwise; and if *S* = 0, it is linear. Directionality was determined using the COUNTIF function in Microsoft Excel 2019 (Microsoft Corporation, Redmond, WA, USA). The PAN was divided into four quadrants, with the image center as the origin. Gaze coordinates were classified into quadrants, and transitions between quadrants were used to further characterize the scan paths. Based on these criteria, eye movement patterns were classified as clockwise (R group), counterclockwise (L group), scattered with few fixations and frequent saccades (S group), and clustered around anatomical landmarks with minimal quadrant transitions (C group).

### 2.7. Saccadic Analysis

Because eye movements involve angular displacement, saccadic amplitude was measured in degrees. Saccades were defined as movements with velocity ≥ 30°/s. The total number of saccades was counted and normalized per minute to account for variations in viewing time.

### 2.8. Ordinal Logistic Regression

Ordinal logistic regression analysis was conducted to evaluate predictors of task performance. The task score (ordinal, 0–5) was the dependent variable, and physiological indices (eye movement, pupil, and blink measures) were included as continuous explanatory variables. The models were fitted using maximum likelihood estimation. The model fit was assessed using Akaike’s Information Criterion (AIC) and McFadden’s pseudo-R^2^.

### 2.9. Statistical Analysis

Student’s *t*-tests were used to examine sex differences in task scores and eye-tracking measures. Chi-squared tests were applied to compare interpretation outcomes across eye movement patterns, followed by residual analysis. The relationship between the task score and viewing time was analyzed using a linear mixed-effects model (LMM), with the score as the dependent variable, viewing time category as a fixed effect, and participant ID as a random intercept.

For comparisons of task scores and eye-tracking indices among eye movement patterns, one-way ANOVA followed by Tukey–Kramer post hoc tests were used for normally distributed data with equal variance. For non-parametric data, Kruskal–Wallis tests followed by Steel–Dwass post hoc comparisons were applied. All analyses were performed using Bell Curve for Excel (ver. 3.22; Social Survey Research Information Co., Ltd., Tokyo, Japan).

LMM and ordinal logistic regression analyses were performed using Python software (version 3.12.7; Python Software Foundation, Wilmington, DE, USA) using the statsmodels library, with the models built using the OrderedModel class. A two-tailed significance level of *p* < 0.05 (two-tailed) was considered.

Group comparisons were conducted using one-way ANOVA. Welch’s ANOVA was applied when the assumption of homogeneity of variance was violated. For non-normally distributed variables, the Kruskal–Wallis test followed by post hoc comparisons using the Dunn–Bonferroni method was performed. LMMs were used to analyze repeated measures across time bins. Effect sizes were reported as Cramer’s V for chi-squared tests and partial eta-squared (η^2^) for ANOVAs, with thresholds interpreted according to conventional guidelines (small, moderate, and large). Statistical significance for all analyses was set at *p* < 0.05.

## 3. Results

### 3.1. Eye Movement Patterns

The eye movement patterns were classified into four groups: clockwise (R; *n* = 16, 29.6%), counterclockwise (L; *n* = 24, 44.4%), scattered (S; *n* = 9, 16.7%), and clustered (C; *n* = 5, 9.3%). No significant sex differences were observed in the distribution of eye movement patterns (χ^2^(3) = 1.33, *p* = 0.72). Representative examples of each pattern are shown in Figure 2. In the R group (Figure 2a), scan paths were characterized by fixations distributed across the entire radiograph and relatively few saccades. An example of the L group is shown in Figure 2b, whereas the S group demonstrated a pattern dominated by saccades with very few fixations (Figure 2c). In the C group, fixation was restricted to specific landmarks (Figure 2d).

### 3.2. Distribution of Scores by Eye Movement Pattern

No sex differences were observed in task scores or eye-tracking measures. The overall mean task score was 2.13 (SD = 1.11, median = 2.0); therefore, participants scoring ≥ 3 points were classified as the high-score group, and those scoring < 3 points as the low-score group. The distribution of task scores based on eye movement patterns is shown in Table 1. The chi-squared test revealed a significant association between group and performance level (χ^2^(3) = 29.66; *p* < 0.001; Cramer’s V = 0.74). The adjusted standardized residuals indicated that the R group contained significantly more participants in the high-scoring group and fewer in the low-scoring group than expected, whereas the L group contained significantly more participants in the low-scoring group and fewer in the high-scoring group. No significant deviations from the expected frequencies were observed in the C and S groups.

### 3.3. PAN Interpretation Results by Eye Movement Patterns

The task outcomes according to the patterns are summarized in Table 2. Chi-squared tests indicated significant group differences in the detection of restorations, dental age, and congenital anomalies (all *p* < 0.001). Residual analysis further revealed that the R group achieved significantly higher detection rates for dental age and congenital anomalies than the L, S, and C groups (both *p* < 0.001). The mean task scores also differed significantly across groups (ANOVA revealed a significant main effect of group [ANOVA, *p* < 0.001], with post hoc comparisons indicating group differences), with the R group scoring higher than the L, S, and C groups (all *p* < 0.001, Tukey–Kramer test). These results indicate that interpretation accuracy and task score differed by scan pattern.

### 3.4. Eye-Tracking Indices by Eye Movement Patterns

The eye tracking indices according to the patterns are presented in Table 3. The S group showed a significantly higher average saccadic velocity and shorter mean fixation duration than the other groups (*p* = 0.010 and *p* = 0.013, respectively; ANOVA). Significant differences were observed between the S and L groups in the number of fixations and total fixation duration (*p* = 0.013 and *p* = 0.026, respectively). Significant differences in blink measurements (blink count, Voluntary blinks, and rapid serial blinks) were found between the R and S groups (*p* = 0.043, *p* = 0.037, and *p* = 0.024, respectively). No significant differences in the pupil area were observed among the four groups (ANOVA, *p* > 0.05).

### 3.5. Saccadic Analysis

Table 4 presents the results of the saccadic analysis. The S group exhibited a significantly greater mean saccadic amplitude than the other groups (*p* = 0.008, Kruskal–Wallis test), whereas the C group showed the smallest amplitudes. The number of saccades per minute was significantly higher in the S group than in the C group (*p* < 0.001).

### 3.6. Ordinal Logistic Regression

To evaluate the primary hypothesis regarding the effect of pupil area on the task score, ordinal logistic regression was conducted, excluding eye movement patterns as a factor. The predictors included total viewing time, mean saccadic velocity, fixation duration, fixation count, pupil area, pupil area during fixation, voluntary blinking, and rapid serial blinks. The model fit indices indicated an acceptable fit (AIC = 139.9; McFadden’s pseudo R^2^ = 0.16). The significant predictors are summarized in Table 5.

The pupil area (coefficient (coef) = −0.625, SE = 0.218, z = −2.87, *p* = 0.004) and blink measures (Voluntary blinks, coef = −0.313, *p* = 0.035; rapid serial blinks, coef = −0.127, *p* = 0.003) were negatively associated with task score.

The pupil area during fixation was positively associated with task score (coef = 0.510, *p* = 0.014).

Total viewing time was not a significant predictor (*p* = 0.310).

### 3.7. Effect of Viewing Time on Task Performance

Finally, the viewing time was categorized into four bins and analyzed using a linear mixed model (Table 6). Task scores increased significantly up to 120 s (0–60 s: mean = 1.9, *p* < 0.001; 61–120 s: mean = 2.44, *p* = 0.022) but did not significantly improve beyond 121 s (121–180 s: mean = 1.6, *p* = 0.463; ≥181 s: mean = 2.0, *p* = 0.900).

### 3.8. The Distribution of Viewing Times Across Eye Movement Patterns

The distribution of viewing times across eye movement patterns is presented in Table 7.

In the R group, the proportion of students with a viewing time of 61–120 s was significantly higher than in the other groups, whereas the proportion with a viewing time of 121–180 s was significantly lower than in the other groups. In the L group, the proportion of students with a viewing time of 121–180 s was significantly higher than in the other groups. No significant differences were observed in the distribution of viewing times between the S and C groups. A chi-squared test of independence confirmed a significant association between eye movement patterns and viewing time (χ^2^(9) = 23.05, *p* = 0.0061, Cramer’s V = 0.49), indicating a moderate effect size.

At the 61–120 s interval, which represented the mode in the R, L, and S groups, ANOVA revealed a significant main effect of group (*p* < 0.001). Post hoc comparisons indicated that the R group achieved significantly higher task scores (3.46 ± 0.66) compared with the L (1.62 ± 0.77) and S (1.80 ± 1.30) groups.

## 4. Discussion

In this study, even when participants were allowed to freely observe the PAN without time restrictions, eye movement patterns similar to those reported in our previous study were identified (R, 29.6%; L, 44.4%; S, 16.7%). The distribution of patterns was consistent with our earlier findings (R, 36.7%; L, 48.3%; S, 15%), suggesting a certain degree of stability in visual perceptual behavior despite differences in viewing time and participants. In addition, we identified a new localized eye movement pattern in which the gaze was concentrated around anatomical landmarks (C pattern: 9.3%). Saccade analysis revealed that the C group exhibited significantly fewer saccades with shorter amplitudes than the other groups, whereas the S group showed a significantly higher saccadic frequency and greater amplitude.

Pannasch et al. [13] reported that under free-viewing conditions with a short viewing duration (6.5 s), eye movements typically shift from ambient (global exploration) to focal (detailed inspection) processing. However, when viewing unfamiliar aerial photographs, they found that the difficulty of information processing led to a prolonged dominance of ambient exploration [14]. The S-pattern, characterized by sparse fixations and frequent large saccades, appears to reflect such a prolonged ambient/global search under increased task difficulty. The blink rate of the S group was significantly higher than that of the other groups. Blink-related measures have been linked to cognitive/attentional state and increased cognitive load or processing difficulty in prior work [15,16,17], and elevated blink activity may also accompany fatigue-related or effortful processing [18]. Therefore, the increased blink activity observed in group S may reflect less stable attentional engagement during interpretation, which is consistent with their shorter fixation durations and higher saccadic frequency. As mixed-dentition PANs show unerupted permanent tooth germs located beneath the primary teeth, students accustomed to adult PANs may have perceived them as less familiar, thereby maintaining global exploration for a longer period.

In contrast, pattern C was characterized by highly localized fixations and reduced saccadic frequency and amplitude from the outset, suggesting a local-first (focal) strategy rather than a typical ambient-to-focal transition. This detail-focused style is consistent with the literature on local processing bias in Autism Spectrum Disorder, including the weak central coherence account [19] and the enhanced perceptual functioning framework [20], although such a bias can also manifest as a cognitive style within nonclinical populations [21]. Consequently, the C pattern, where the gaze was narrowly directed toward landmarks, may represent a form of local search strategy, in contrast to a global search, which involves scanning the entire visual field. This pattern could reflect a field-dependent or detail-oriented exploratory tendency, potentially associated with individual differences in visual search styles. Furthermore, individual differences in cognitive capacity and attentional control may influence scan pattern variation, which is consistent with evidence that cognitive traits modulate visual exploration strategies [22]. Because ASD-related traits or clinical characteristics were not assessed, this comparison is intended as a conceptual analogy for local processing and does not imply clinical features in the participants.

Analysis of viewing time showed that R-group students were more likely to complete the task within 61–120 s and less likely to exceed 121 s, whereas L-group students tended to observe longer than 121 s. No significant differences were noted for the S or C groups. These findings indicate that the R-pattern may be associated with a more globally organized scan path and higher task scores in this single-image setting.

Importantly, longer viewing time did not necessarily translate into higher task scores. Both ordinal logistic regression and LMM analyses demonstrated that viewing times beyond 121 s were not associated with further improvements in task scores. This suggests that prolonged inspection, rather than enhanced task-related reasoning, may reflect an increased memory load in students who require more time to encode and report findings.

The pupillometric analyses supported these interpretations. Students in the high-score group exhibited a smaller mean pupil area across the entire viewing time but larger dilation during fixations on task-relevant regions (i.e., the pre-specified items). Ordinal logistic regression analysis revealed that a smaller mean pupil area across the overall viewing time, combined with a larger pupil area specifically during fixations, was significantly associated with higher task scores. In our previous study using a strict 1 min viewing time limit, a larger pupil area in students with higher task scores may have reflected higher overall cognitive load under time pressure. In the present free-viewing design, separating overall (tonic) pupil area from fixation-related (phasic) pupil area revealed a different pattern, highlighting the importance of task constraints and measure definition when interpreting pupillary indices. This tonic–phasic dissociation may indicate a more selective strategy in which cognitive load is kept relatively low during global scanning but increases when task-relevant regions are fixated for detailed evaluation. This pattern is consistent with task-evoked pupillary responses [23,24] and may suggest more selective allocation of cognitive resources, characterized by lower tonic load and larger phasic dilation during task-relevant fixations. Similar results have been reported in expert populations, who maintain a stable baseline pupil area with marked phasic dilation during task-relevant events [11,25]. In contrast, students in the lower-score group displayed persistently larger pupil areas, suggesting a sustained cognitive load throughout the viewing time. This interpretation is consistent with evidence that high memory demands and cognitive load elicit sustained pupil dilation, often associated with poorer task performance [26,27].

Taken together, these findings were broadly consistent with our initial hypotheses. In this single-image task, higher task scores were associated with a higher prevalence of a clockwise scan pattern and completion of viewing within 120 s, whereas viewing beyond 121 s was not associated with further improvement in task score. In addition, the observed tonic–phasic pupil pattern suggests that higher performance may be related to more selective allocation of cognitive resources during task-relevant fixations. Given the exploratory single-image design with pre-specified items, these findings should be interpreted as task-specific associations and warrant replication using multiple images and coverage-based metrics to assess robustness and generalizability. In addition, some clinicians/researchers assess panoramic radiographs by compartmentalizing the image into anatomical zones that include peripheral regions such as the TMJ, ramus–spine, and hyoid areas [28,29]. Because our representative scan paths primarily illustrate central viewing behavior, peripheral zones may have been undersampled in some students; therefore, future studies should incorporate zone-based coverage metrics to quantify scanning completeness across the entire panoramic field [29]. In this study, multiple scan patterns (counterclockwise, scattered, and concentrated) were observed, despite students having received standardized instruction in the FDI-based clockwise viewing method. This suggests that visual strategies are not solely determined by instructional direction and may reflect individual perceptual tendencies. Furthermore, cognitive load is reflected not only in pupil area but also in other oculomotor indices. Microsaccadic activity has been linked to mental effort [30], indicating that incorporating microsaccade metrics could complement pupil-based assessments in future studies.

Collectively, these results highlight the potential value of objective oculomotor metrics for characterizing task-specific performance and for guiding future research in dental radiology education.

From an educational perspective, the present findings may provide preliminary support for using eye-movement and pupillometry metrics as objective indices for hypothesis generation in dental radiology training research. Future multi-image studies with verified findings are warranted to examine whether feedback based on these metrics can improve task performance and scan coverage, thereby potentially informing the development of evidence-based educational strategies.

## 5. Limitations

This study had several limitations. Firstly, although pupillary responses were interpreted as indicators of cognitive load, pupil area is also influenced by autonomic arousal. Because physiological indices of sympathetic activity, such as the galvanic skin response (GSR) and heart rate variability (HRV), were not measured in this experiment, the contribution of autonomic factors cannot be excluded. Future studies should incorporate simultaneous GSR/HRV recordings along with eye tracking. Studies may benefit from including microsaccade measurements, given that microsaccadic rate is sensitive to cognitive load, thereby enabling more comprehensive assessment while dissociating cognitive and autonomic influences during radiographic interpretation.

Secondly, individual differences in cognitive style were not assessed. Visual search strategies may be associated with field dependence or field independence, which were not evaluated in this study. Incorporating validated measures of cognitive style in future studies will help clarify how perceptual strategies interact with eye movement patterns, pupillary responses, and task performance.

Thirdly, the participants’ baseline diagnostic knowledge was assumed to be equivalent because all students had passed the nationwide CBT required for clinical training. Although a formal calibration pre-test was not conducted, we avoided administering such a test because it could have influenced scanning behavior and introduced instructional bias. Future studies should incorporate a pre-assessment to verify competency homogeneity and reduce the variation associated with prior experience.

Finally, the study was limited by the use of a single mixed-dentition PAN and a fixed cohort size. Focusing on a single radiograph and a limited set of pre-specified diagnostic traits may capture only a fraction of the diagnostic process and may introduce bias. Therefore, the present findings cannot be generalized to represent dental students’ overall diagnostic competence and should be interpreted as an exploratory analysis of eye-movement dynamics under a specific, single-image condition. In addition, because tooth-by-tooth coverage and area-of-interest (AOI)-based fixation were not quantified across all anatomical regions, it remains unclear whether regions without direct fixation were processed via peripheral vision or overlooked. Future studies should expand the sample size and incorporate multiple radiographic images with a broader range of verified findings, together with AOI-based coverage metrics, to improve reproducibility and generalizability.

## 6. Conclusions

In this exploratory single-image task, specific scan-path characteristics and pupillary/blink dynamics were associated with higher task scores during interpretation of a mixed-dentition panoramic radiograph. However, these associations should be interpreted with caution because they reflect performance within a specific, pre-specified-item task and do not indicate overall diagnostic competence or superiority across diverse clinical scenarios. Notably, a smaller mean pupil area across the overall viewing period and a larger pupil area during fixations on task-relevant regions were associated with higher task scores, consistent with differential tonic versus phasic pupillary responses. Replication using multiple panoramic images with a broader range of verified findings, together with coverage-based metrics, is required to evaluate the robustness, generalizability, and potential educational implications of these findings.

## Figures and Tables

**Table 1:** Distribution of scores by eye movement pattern.

	R (*n* = 16)	L (*n* = 24)	S (*n* = 9)	C (*n* = 5)
High-score group (%)	15 (93.8) **	5 (20.8)	1 (11.1)	0 (0.0)
Low-score group (%)	1 (6.2)	19 (79.2) **	8 (88.9)	5 (100.0)

** *p* < 0.001 (chi-squared test); R: clockwise; L: counterclockwise; S: scattered/saccadic dominant; C: restricted/clustered gaze pattern. Groups were defined based on the task score (≥3 vs. <3), not as a measure of overall diagnostic competence.

**Table 2:** Test image interpretation results by eye movement pattern.

	R (*n* = 16)				L (*n* = 24)				S (*n* = 9)				C (*n* = 5)				*p*-Value *
	Correct (%)		Incorrect (%)		Correct (%)		Incorrect (%)		Correct (%)		Incorrect (%)		Correct (%)		Incorrect (%)		
Supernumerary teeth	3	(18.8)	13	(81.2)	2	(8.3)	22	(91.7)	1	(11.1)	8	(88.9)	0	(0.0)	5	(100.0)	0.62
Space maintainer	16	(100.0)	0	(0.0)	23	(95.8)	1	(4.2)	9	(100.0)	0	(0.0)	5	(100.0)	0	(0.0)	0.74
Restoration	12	(75.0)	4	(25.0)	5	(20.8)	19	(79.2)	1	(11.1)	8	(88.9)	0	0.0	5	(100.0)	<0.001
Dental age	15	(93.8)	1	(6.2)	8	(33.3)	16	(66.7)	2	(22.2)	7	(77.8)	2	(40.0)	3	(60.0)	<0.001
Congenital anomalies (present/absent)	10	(62.5)	6	(37.5)	2	(8.3)	22	(91.7)	0	(0.0)	9	(100.0)	0	(0.0)	5	(100.0)	<0.001
Task score (points) ^†^	Mean (SD)				Mean (SD)				Mean (SD)				Mean (SD)				
	3.50 (0.63) ^a^				1.67 (0.76) ^b^				1.44 (0.73) ^b^				1.40 (0.55) ^b^				<0.001

R: clockwise; L: counterclockwise; S: scattered/saccadic dominant; C: restricted/clustered gaze pattern; SD: standard deviation. * Chi-squared test. ^†^ One-way ANOVA with Tukey–Kramer post hoc tests. Superscript letters indicate significant differences within each row; means sharing a letter are not significantly different. Task score was the number of correctly identified pre-specified items (0–5), selected for ground-truth verification in a single-image design; other potential findings were not scored.

**Table 3:** Eye movement measures among the four groups.

	R (*n* = 16)		L (*n* = 24)		S (*n* = 9)		C (*n* = 5)		
	Mean	(SD)	Mean	(SD)	Mean	(SD)	Mean	(SD)	*p*
Total viewing time (s)	85.3	(28.5)	109.8	(48.4)	78.6	(51.6)	90.9	(64.4)	0.231
Average gaze velocity (deg/s) *	50.6	(56.3) ^a^	45.3	(26.4) ^a^	89.7	(28.9) ^b^	24.4	(7.9) ^a^	0.010
Mean fixation duration (ms) *	50.2	(13.2) ^a^	50.3	(15.5) ^a^	33.8	(0.6) ^b^	53.9	(15.6) ^a^	0.013
Number of fixations (n) *	299.4	(148.6) ^ab^	416.3	(305.4) ^a^	94.8	(62.0) ^b^	397.2	(345.0) ^ab^	0.013
Total fixation duration (s) *	16.4	(10.9) ^ab^	23.3	(20.5) ^a^	3.2	(2.2) ^b^	25.5	(28.0) ^ab^	0.026
Pupil area (π·dot^2^)	46.9	(8.6)	51.5	(9.2)	49	(11.9)	54.4	(14.2)	0.381
Pupil area during fixation (π·dot^2^)	47.1	(8.4)	51.2	(9.0)	50.2	(12.1)	53.1	(15.7)	0.549
Spontaneous blinks (times) *	66.8	(45.7) ^a^	92.7	(59.7) ^ab^	119.2	(75.4) ^b^	41.6	(33.4) ^ab^	0.043
Voluntary blinks (times) ^†^	1.4	(1.7) ^a^	1.9	(2.4) ^ab^	5.1	(5.6) ^b^	2.2	(3.5) ^ab^	0.021
Rapid serial blinks (times) *	8.8	(8.7) ^a^	14.4	(11.0) ^a^	21.7	(16.8) ^b^	5.6	(4.7) ^a^	0.024

R: clockwise; L: counterclockwise; S: scattered/saccadic dominant; C: restricted/clustered gaze pattern; SD: standard deviation. * Variables analyzed with ANOVA (Welch’s or standard as appropriate); spontaneous blink only by standard ANOVA. ^†^ Variable analyzed with Kruskal–Wallis and Steel–Dwass post hoc tests. Superscript letters indicate group differences within each row. Means sharing the same letter are not significantly different (*p* < 0.05).

**Table 4:** Saccade analysis by eye movement pattern.

	R (*n* = 16)		L (*n* = 24)		S (*n* = 9)		C (*n* = 5)		*p*-Value
	Mean	(SD)	Mean	(SD)	Mean	(SD)	Mean	(SD)	
Total amplitude (deg)	4290.6	(3224)	5115.9	(3486)	5889.7	(3801)	2069.2	(1474)	0.198
Average saccade amplitude (deg) *	2.4	(2.6) ^a^	2.3	(1.7) ^a^	5.2	(3.7) ^b^	1.1	(0.5) ^a^	0.008
Number of Saccades (n/min) *	396.6	(193) ^a^	345.9	(209) ^a^	784	(331) ^b^	288.4	(132) ^a^	<0.001

R: clockwise; L: counterclockwise; S: scattered/saccadic dominant; C: restricted/clustered gaze pattern, SD: standard deviation. * Average saccade amplitude was analyzed using Welch’s ANOVA, and the number of saccades was examined using standard ANOVA. Superscript letters indicate differences among the four groups within each row. Values sharing the same superscript letter indicate no significant differences, whereas different letters indicate significant differences according to post hoc comparisons (*p* < 0.05).

**Table 5:** Ordinal logistic regression analysis of eye movement and pupillary measures.

Variable	Coefficient	Standard Error (SE)	z	*p*-Value	95% CI	
					[2.5%]	[97.5%]
Total viewing time (s)	0.0124	0.012	1.015	0.31	−0.012	0.036
Average amplitude (deg)	0.0202	0.012	1.759	0.079	−0.002	0.043
Mean fixation duration (ms)	0.0102	0.027	0.376	0.707	−0.043	0.063
Fixation count (number)	−0.00002	0.002	−0.009	0.993	−0.004	0.004
Pupil area (π·dot^2^)	−0.6252	0.218	−2.872	0.004	−1.052	−0.199
Pupil area during fixation (π·dot^2^)	0.5103	0.208	2.45	0.014	0.102	0.918
Voluntary blinks (times)	−0.3127	0.148	−2.107	0.035	−0.604	−0.022
Rapid serial blinks (times)	−0.1266	0.043	−2.952	0.003	−0.211	−0.043

Results of the ordinal logistic regression analysis of eye movement and pupillary measures. The table presents regression coefficients, standard errors (SE), z-values, and corresponding *p*-values with 95% confidence intervals (CI). Significant predictors of outcome variables included pupil area (*p* = 0.004), pupil area during fixation (*p* = 0.014), voluntary blink frequency (*p* = 0.035), and rapid serial blink frequency (*p* = 0.003).

**Table 6:** Mean (±SD) of outcome across time bins and LMM results.

Time Bin (s)	Mean	(SD)	*p*-Value
0–60	1.90	(0.88)	<0.001
61–120	2.44	(1.24)	0.022
121–180	1.60	(0.70)	0.463
181+	2.00	(1.41)	0.900

Mean values and standard deviations (SD) of the outcome variable across time bins (0–60 s, 61–120 s, 121–180 s, and ≥181 s). Linear mixed model (LMM) results with corresponding *p*-values are shown. Significant effects were detected at 0–60 s (*p* < 0.001) and 61–120 s (*p* = 0.022).

**Table 7:** Distribution of viewing times across eye movement patterns.

Time	R (*n* = 16)	L (*n* = 24)	S (*n* = 9)	C (*n* = 5)
0–60 s	3	2	3	2
61–120 s	13 *	13	4	1
121–180 s	0 *	8 *	0	2
181+ s	0	1	1	0

* *p* < 0.01 (chi-squared test). R: clockwise; L: counterclockwise; S: scattered/saccadic dominant; C: restricted/clustered gaze pattern.

## Data Availability

The original data generated in this study are available from the corresponding author upon reasonable request. Due to privacy and ethical restrictions, the data are not publicly available.

## Abbreviations

The following abbreviations are used in this manuscript:

PAN	Panoramic radiograph
CBT	Common Achievement Tests
AIC	Akaike’s information criterion
ANOVA	Analysis of variance
GSR	Galvanic skin response
HRV	Heart rate variability
